# The Tolerance Characteristics of Resident Fish in the Upper Yangtze River under Varying Gas Supersaturation

**DOI:** 10.3390/ijerph16112021

**Published:** 2019-06-06

**Authors:** Qianfeng Ji, Shudan Xue, Quan Yuan, Yuan Yuan, Yuanming Wang, Ruifeng Liang, Jingjie Feng, Kefeng Li, Ran Li

**Affiliations:** 1 State Key Laboratory of Hydraulics and Mountain River Engineering, Sichuan University, Chengdu 610065, China; qianfengji@126.com (Q.J.); xxueshudan@126.com (S.X.); yuanq1992@126.com (Q.Y.); ruifengliangscu@126.com (R.L.); fengjingjie@scu.edu.cn (J.F.); kefengli@scu.edu.cn (K.L.); liran@scu.edu.cn (R.L.); 2 Power China Kunming Engineering Corporation Limited, Kunming 650051, China; yuanyuankm92@163.com

**Keywords:** total dissolved gas supersaturation, upper Yangtze River, resident fish, tolerance characteristics, gas bubble disease

## Abstract

In circumstances where total dissolved gas (TDG) levels are variable, the peak TDG and duration are expected to be the dominant drivers of fish survival. Focusing on the peak TDG and duration in natural rivers, a laboratory experiment and field experiments in the upper Yangtze River were conducted with Prenant’s Schizothoracin (*Schizothorax prenanti*), a rare species inhabiting the upper Yangtze River, to examine the tolerance characteristics of fish under varying gas supersaturation levels. The results of the field experiments showed that TDG supersaturation in natural rivers changed greatly during the flood period due to reservoir regulation. The survival of fish was affected by TDG levels, water depth and TDG fluctuation range. A high TDG level, and shallow compensatory water depth caused fish mortality in the field experiment to be higher in September than in July. The results of the laboratory experiment showed that fish tolerance was lower under fluctuating TDG supersaturation than under constant TDG supersaturation. The tolerance of fish to TDG supersaturation varied depending on peak TDG and duration. Under the fluctuation range of 115–125%, fish survival in the 6 h–6 h cycle was significantly different from that in the 8 h–8 h cycle. The fluctuation cycle did not affect fish survival at the fluctuation range of 110–130%. Intermittent lower TDG supersaturation does not significantly increase the tolerance of fish. This study revealed the tolerance characteristics of resident fish in the upper Yangtze River to TDG supersaturation, which provides a reference to the ecological operation of reservoirs and may contribute to the protection of aquatic organisms.

## 1. Introduction

Total dissolved gas (TDG) supersaturation is a physical condition in which the pressures of atmospheric gases in a solution exceed the barometric pressure [1] and is a common phenomenon in nature [2]. Aquatic organisms are susceptible to developing gas bubble disease (GBD) and even suffering mortality when river ecosystems experience TDG supersaturation [3,4,5]. The causes of TDG supersaturation in water include photosynthesis [6], ocean wave action [7], temperature [8,9,10], falling water [11], and dam discharge [12], among other factors. TDG supersaturation caused by dam discharge has serious adverse impacts on fish downstream [13]. The large amount of flow discharging from a dam carries a considerable mass of air into the downstream energy-dissipation pool during the flood season. Under the action of hydrostatic and hydrodynamic pressure, the solubility of gases in the pool is much greater than that under normal pressure, leading to TDG supersaturation downstream of the dam. TDG supersaturation is released much more slowly in natural rivers, and its side effects on aquatic organisms extend over a large area and last for a long period of time [14]. In Norway [15], the United States [16], Canada [17] and China [18], a large number of dead fish have been found downstream of dams due to TDG supersaturation. The influence of TDG supersaturation caused by flood discharge on river ecosystems has become an environmental issue of global concern [19,20,21].

The Yangtze River basin is the most developed area in China in terms of freshwater fisheries. The fishery output of the Yangtze River accounts for approximately two-thirds of the fishery output of China and plays a crucial role in the Chinese fishing industry [22]. The upper Yangtze River has many tributaries varying in topography and flow pattern, which provide rich and diverse habitats for fish. There are many rare and endemic species in this region [23]. Therefore, a rare and endemic fish reserve has been established in the upper Yangtze River, which is extremely important for the protection of global biodiversity [24]. With the energy structure adjustment and energy demand growth in China, a large number of high dams in the upper Yangtze River are being planned, designed, or constructed or are already in operation. High dams provide great benefits in terms of flood control, power generation, irrigation and shipping. However, these dams have also caused a series of environmental problems [25]. TDG supersaturation in the upper Yangtze River has recently received much attention from society. According to observations downstream of dams located in the Yangtze River (such as the Three Gorges, Gezhouba, Ertan, Zipingpu, Gongzui, and Tongjiezi Dams, among others), TDG supersaturation is widespread and extremely harmful to fish in this area [26,27]. During the flood discharge of Xiluodu Dam in 2014, approximately 40 tons of fish died downstream of the dam, which aroused great concern from various groups [28]. TDG supersaturation caused by flood discharge from high dams in the upper Yangtze River has seriously threatened the aquatic biodiversity and fishery security in this area. As noted by Chinese President Xi Jinping, for the long-term interests of the Chinese nation, the restoration of the Yangtze River environment should be prioritized, and the focus should be on the protection of the Yangtze River rather than on overdevelopment. However, to date, there has been no evidence that China will slow the pace of hydropower development in the upper Yangtze River [29]. Thus, TDG supersaturation will continue to be a major problem and will need to be solved in the future.

The tolerance of many fish, such as Prenant’s Schizothoracin (*Schizothrorax prenanti*), David’s Schizothoracin (*Schizothorax davidi*), Chinese sucker (*Myxocyprinus asiaticus* Bleeker), Rock carp (*Procypris rabaudi* Tchang), and Silver carp (*Hypophthalmichthys molitrix*), to supersaturated TDG in the Yangtze River basin has been studied. These studies have shown that the tolerance of fish is related not only to the supersaturated TDG level, but also to water temperature, sediment content, and body size [12,30,31,32,33,34,35,36,37,38,39]. Previous studies of fish tolerance characteristics focused on a constant TDG supersaturation level and a constant compensation depth, and most of them were conducted only in the laboratory. However, the discharge mode and discharge flow of hydropower projects frequently change during the flood season, which significantly affects the level of TDG downstream and causes TDG supersaturation to fluctuate. The effects of exposure to gas-supersaturated waters stem directly from physical processes driven by the level of gas supersaturation. Thus, in circumstances where the TDG levels are variable, the peak and duration of the peak are expected to be the dominant drivers of survival. It is necessary to study the tolerance characteristics of fish under variable TDG supersaturation levels, which can provide a reference for hydropower operation and protection of the environment.

Prenant’s Schizothoracin (*Schizothorax prenanti*), a unique economic benthic fish in China, is widely distributed in the upper Yangtze River basin. With the implementation of a series of hydropower engineering projects in the upper Yangtze River, the survival of this species has been seriously threatened [40]. This paper used Prenant’s Schizothoracin as the research object in a laboratory experiment and field experiments in the upper Yangtze River and revealed the tolerance characteristics of endemic fish under variable TDG supersaturation levels. The results provide a reference for ecological hydropower operation and will contribute to the preservation of aquatic organisms.

## 2. Materials and Methods

### 2.1. Field Experiments

**Figure 1:**
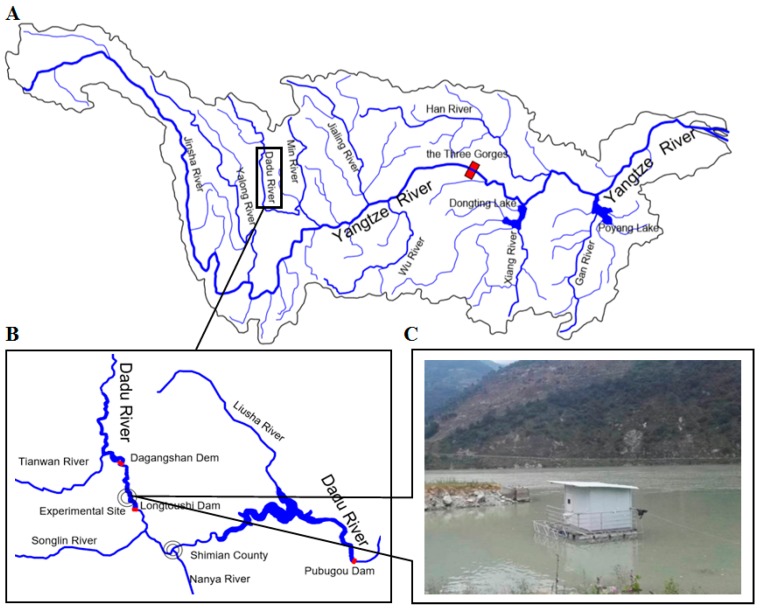
The experimental site during July and September 2017, which is located on the right bank of the Longtoushi Reservoir in the middle Dadu River, a tributary of the upper Yangtze River (**A**,**B**). (**C**) is a photograph of the study site and was taken by author Yuanming Wang.

The experimental site is located in the Dadu River, a tributary of the upper Yangtze River. The site is 1.6 km downstream of the Dagangshan Dam and 3.9 km upstream of the Longtoushi Dam (Figure 1). The Dagangshan Dam is a daily regulating reservoir with a total storage capacity of 742 million cubic meters and a regulating storage capacity of 117 million cubic meters [41]. The flood discharge facilities of this dam consist of four deep holes and an open-type tunnel. Floodwater spilled from the deep holes jets into a plunge pool downstream or from open-type tunnel jets into a scouring pit. The discharge volume of the tunnel ranges from 1838 to 3352 m^3^/s, and the maximum discharge flow of a single deep hole is 1303 m^3^/s. The Longtoushi hydropower station is the 15th cascade power station among the 22 in the Dadu River, and it is also a daily regulating reservoir [42]. This dam has a normal water level of 955 m, a total storage capacity of 134.7 million cubic meters, and a regulating storage capacity of 0.167 billion cubic meters.

The field experiment was conducted in July and September 2017. During the experiment, the level of TDG supersaturation was automatically recorded by a Point Four Tracker (Coquitlam, British Columbia) with a measurement range of 0–200% and an accuracy of 1%, which was calibrated before the experiment. The water depth of the experimental site was measured by a DT110 instrument (DEDUYIQI, China) with a measurement range of 0–10 m and an accuracy of 0.01 m.

**Figure 2:**
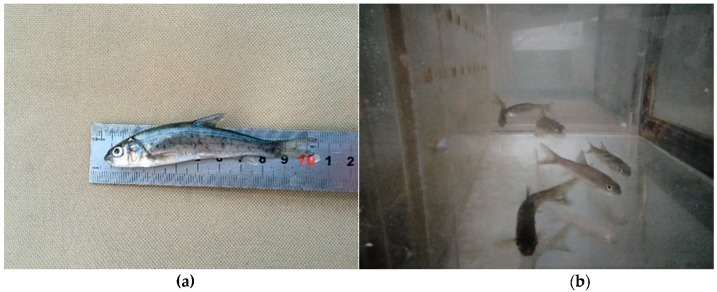
Picture of Prenant’s Schizothoracins used in the present experiment. (**a**) is a dead fish in the experiment, and (**b**) is fish in total dissolved gas (TDG) supersaturated water.

One-year-old Prenant’s Schizothoracins provided by the Fisheries Institute of the Sichuan Academy of Agricultural Sciences were used in the experiment (Figure 2). In July, a total of 60 fish (weight: 9.05 ± 3.45 g, length: 10.2 ± 1.1 cm) were used to conduct the field experiment, which began at 10 am on July 4 and lasted 5 days. In September, 80 fish (weight: 10.35 ± 2.81 g, length: 10.3 ± 1.6 cm) were used to conduct a field experiment from 7 am on September 11 to 9 am on September 16. In addition, 20 experimental fish were selected and kept in a saturated-equilibrium water tank as the control group.

Two identical cages (100 cm × 60 cm × 300 cm in size) with a steel structure and nylon netting were used to conduct the experiment with juvenile fish. The cages were suspended from a platform floating on the river (Figure 1C) and were completely submerged in the river when the water depth was greater than 1 m and partly submerged when the water depth was approximately 1 m. The experimental fish were transported to the site before the experiment, and they were kept in saturated-equilibrium water (dissolved oxygen (DO): 5.81–8.02 mg/L, TDG: 73–100%) for 24 h. During the experiment, dead experimental fish were regularly removed from the cages, and the time of death of the individuals was recorded. Furthermore, the lengths of the tested fish were measured by a tape measure, and their weights were measured by an electronic scale.

### 2.2. Laboratory Experiment

The laboratory experiment used healthy one-year-old juvenile Prenant’s Schizothoracins with a length of 7.4–14.0 cm and a weight of 4.0–27.1 g, provided by the Fisheries Institute of the Sichuan Academy of Agricultural Sciences. Before the experiment, the fish were kept in saturated-equilibrium water (DO: 5.1–6.7 mg/L, temperature: 17.8–19.7 °C, TDG: 98–100%) for 3 days of acclimation, and then randomly selected for laboratory experiments.

The laboratory experiment was conducted with TDG supersaturation generation equipment, which was described in detail by Wang et al. [43]. The level of TDG was measured by a Point Four Tracker, which was calibrated before the experiment. The laboratory experiment consisted of two subtests: a constant TDG test and a fluctuant TDG test. According to the field experiment, the water depth in the river was more than 0.3 m. The water depth in the laboratory experiment was set to 35 cm, and the water temperature was controlled at 17–19 °C. At the beginning of each test, the fish were first introduced into the experimental tank with TDG-supersaturated water. Dead fish were immediately removed during the test process. The time of fish death and the characteristics of the dead fish were recorded at the same time. The lengths and weights of dead fish were recorded using a ruler and an electronic balance, respectively.

In the constant TDG test, the level of TDG was constant. Two cases, namely, one with a TDG level of 115% (CT-1) and one with a TDG level of 120% (CT-2), were used in this test. The test lasted for 72 h, and 20 fish were tested in each case.

For the fluctuant TDG test, the level of TDG repeatedly changed from a low level to a high level, and each transition of TDG supersaturation occurred within ten minutes Two low levels (110 and 115%) and three high levels (120, 125 and 130%) were used in this test. Three TDG cycles (6 h−6 h, 8 h−8 h and 12 h−12 h) were chosen according to the fluctuation of TDG observed in the field. A total of 9 cases (combinations of different levels and cycles) were used in this test (Table 1). All cases were conducted for 72 h, and 20 fish were tested in each case. In addition, 20 experimental fish were selected and kept in a saturated-equilibrium water tank as the control group.

### 2.3. Data Analysis

In the field and laboratory experiments, the mortality of the experimental fish was calculated as follows:

(1) $$P=\left(n_{i}/N_{i}\right)\times 100\%,$$

where *P* is the mortality of the test fish, *n_i_* is the number of dead fish in each case, and *N_i_* is the total number of tested fish.

The median lethal time (LT50) was used to evaluate the tolerance of fish in the context of laboratory experiments. This metric refers to the time when the fish mortality reaches 50% under certain conditions and is calculated by Miller’s and Sunter’s graphical methods [44]. Survival analysis was used to evaluate the viability of experimental fish in the laboratory experiment. The survival rate of experimental fish in each case was calculated as follows [45]:

(2) $$S\left(t\right)=P\left(t<T\right)=\prod_{t\left(r\right)\leq t}\frac{n-r}{n-r+1},$$

where *S*(*t*) is the survival function in each case, *P* is the cumulative survival rate, *t* is the survival time of each fish, *n* is the number of surviving experimental fish at time *t*, r is the number of surviving experimental fish that were censored at time *t*, and *T* is the total experiment time. The log-rank Mantel–Cox test was used to compare survival between each pair of cases.

The incidence of signs of GBD was also evaluated in this study and was calculated as follows:

(3) $$P_{d}=\left(\frac{n_{j}}{N_{j}}\right)\times 100\%,$$

where *P_d_* is the rate of occurrence of GBD symptoms, *n_j_* is the number of symptoms occurring in dead fish in each case, and *N_j_* is the total number of dead fish in each case.

## 3. Results

### 3.1. Field Experiments

**Figure 3:**
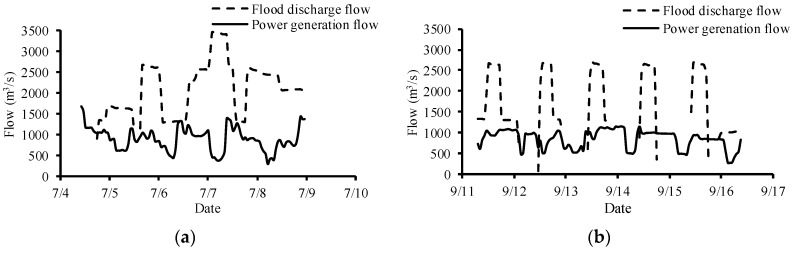
Flow discharge from the Dagangshan hydropower station in July (**a**) and September (**b**).

**Figure 4:**
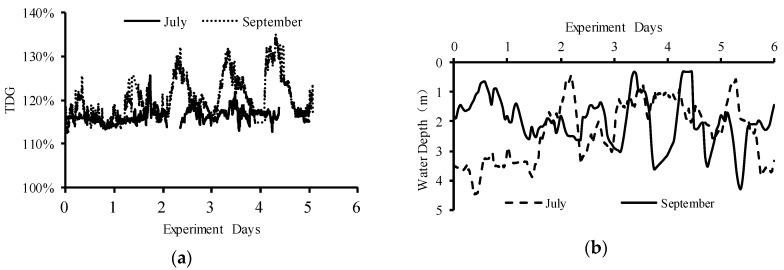
TDG supersaturation level (**a**) and water depth (**b**) at the experimental site. TDG data are missing in two periods in July due to powering off the Point Four Tracker.

**Figure 5:**
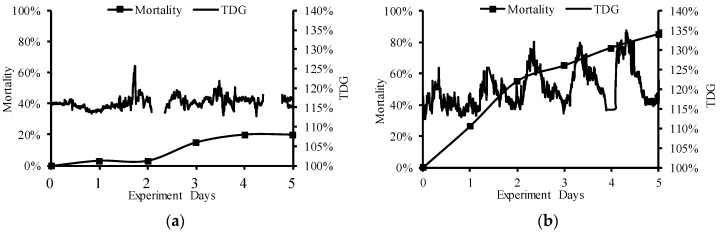
Mortality at the experimental site during the two field experiments, (**a**,**b**) represent the mortality in July and September, respectively. TDG data are missing in two periods in July due to powering off the Point Four Tracker.

As shown in Figure 3, the flow discharge from Dagangshan Dam was significantly different between July and September. The flow process changed periodically during the September field experiment. The average flow was 2755 m^3^/s in July and 2012 m^3^/s in September. The level in July was relatively stable, although TDG data were missing in two periods due to powering off the Point Four Tracker. During the experiment in September, the TDG level periodically fluctuated, and the fluctuation cycle of TDG supersaturation was roughly the same as that of the flood discharge (Figure 4). The average value of TDG was 116% in July and 120% in September. The water depth ranged from 0.43 to 4.46 m in July and from 0.30 to 4.29 m in September. There were seven times in July and four times in September when the water depth was less than 1 m. The cumulative time during which the water depth was below 1 m was 10.33 h in July and 19.98 h in September. The longest duration when the water depth was lower than 1 m was 4.33 h in July and 7 h in September. The mortality of juvenile fish in July reached 20%, while the fluctuating TDG supersaturation in September caused severe mortality, which reached 85% (Figure 5). Among the dead fish, 75% died within the first three days of the experiment in July, and 76.5% died within the first three days of the experiment in September. No fish died at control groups both in July and September.

### 3.2. Laboratory Experiment

**Figure 6:**
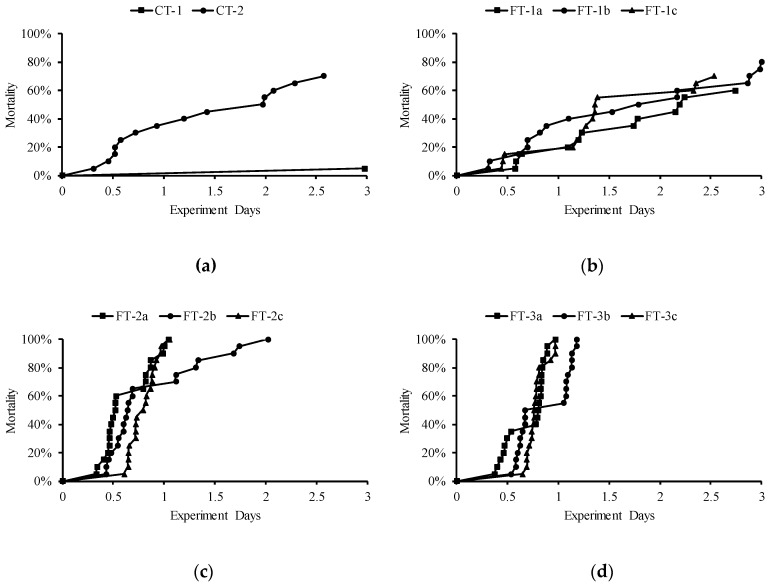
The mortality of fish in each case, (**a**–**d**) represent the mortality in CT, FT-1(a–c), FT-2(a–c), and FT-3(a–c), respectively.

The mortality in each case is shown in Figure 6. In the constant TDG test, fish survived well in case CT-1, in which only one fish died (at 71.5 h), and the final mortality of fish in case CT-2 reached 70%. In the cases FT-1(a–c), where the level of TDG fluctuated from 100 to 120%, the final mortalities were 60, 80, and 70%, respectively. The mortalities in the other cases, where the levels of TDG fluctuated from 115 to 125% or 110 to 130%, reached 100%. No fish died in the control group. The prevalence of GBD in the dead fish is shown in Table 2. Bubbles were found in over 60% of pectoral fins, over 70.0% of dorsal fins and over 65.0% of caudal fins in each case.

Since the fish in CT-1 survived well and only one death was observed during the experiment, the LT50 was not calculated in this case. The LT50 values of fish in the other cases are shown in Table 3. The LT50 ranged from 13.6 (FT-2a) to 53.8 h (FT-1a).

**Figure 7:**
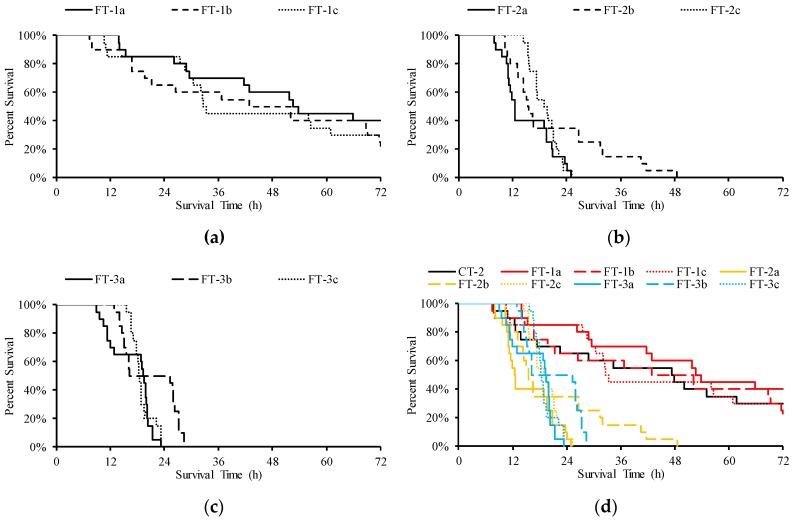
The result of survival analysis for each test, (**a**–**c**) are the percent survival in FT-1(a–c), FT-2(a–c), and FT-3(a–c), respectively, and (**d**) is the percent survival in all cases

The survival analysis function for each test is shown in Figure 7, and the differences between the tests are shown in Table 4. The differences between CT-2 and FT-1(a–c) were not significant, while CT-2 and FT-1(a–c) were significantly different from the other cases.

## 4. Discussion

Total pressure increases with water depth and increases the amount of atmospheric gas held in the water. Compensation by the increase in total pressure protects fish from developing GBD in supersaturated water. The compensation rate is approximately 10% saturation per meter of water depth [46], allowing fish to avoid the threat of TDG supersaturation. However, to guarantee that the flood period can be safely passed, the Longtoushi Dam always operates at a relatively lower water level during the period of Dagangshan Dam discharge. The minimum water depth was 0.43 m in July and 0.3 m in September, which caused fish to receive little compensation from water depth.

The two field experiments showed that the mortality of the fish in September was much higher than that in July. The main reason for this difference was that the peak value was higher and the water depth conditions were worse in September than in July. In contrast to fish mortality in the laboratory experiment, that in the two field experiments did not reach 100% after 120 h of TDG exposure. Compared with the water depth of 35 cm used in the laboratory experiment, the water depth in the field experiments had a greater compensatory effect on the fish.

Bubbles were obvious in fish when they were exposed to TDG-supersaturated water. Under TDG supersaturation, gas can easily enter the fish body through the gills and gradually form a gas embolism, which can result in bubbles, bleeding, and even mortality [47]. Fins are the locomotive organs of fish. The main functions of the pectoral fins are to advance the body, control direction and brake. The dorsal fin is used to balance the fish so that it does not roll over during swimming. The caudal fin can push the body forward and control the swimming direction [48]. When fish are exposed to TDG-supersaturated water, the congestion of the fins has a serious impact on the normal swimming behaviour of the fish. In the laboratory study, many bubbles adhered to the fins of experimental fish, and the fish gradually lost their balance with increasing exposure time. Side swimming and spinning also occurred. For some of the fish that died, the body was curved and stiff.

Many studies have shown that fish tolerance to supersaturated TDG varies with species. As shown in Table 5, 7-day-old David’s Schizothoracins had the strongest tolerance to supersaturated TDG, while the tolerance of Rock carp was weaker than that of other species. In the present study, the LT50 of Prenant’s Schizothoracin exposed to 120% TDG-supersaturated water was 37.7 h, which was longer than the values obtained in previous studies.

Discharge flow is linearly related to TDG supersaturation [21,27]. Under the same amount of discharge, different flow schemes will lead to different TDG supersaturation processes. Compared with the tolerance of fish in the constant TDG test, that in the fluctuant TDG test was significantly lower. In the FT-2 cases, the average TDG value was 120%, with a fluctuation range of ±5%. The fluctuation cycles of FT-2(a–c) were 6 h–6 h, 8 h–8 h, and 12 h–12 h, respectively, and the LT50 of fish in these cases was reduced by 63.7, 52.9 and 50.4%, respectively, compared with that in the 120% constant TDG test. When the TDG supersaturation fluctuated ±10% in the FT-3 cases, the LT50 of fish in the 6 h–6 h, 8 h–8 h and 12 h–12 h cycles was reduced by 58.2, 48.5 and 50.9%, respectively.

In the FT-1 cases, where the average TDG levels were 115%, the final mortality of fish reached 60, 80 and 70% under the fluctuation cycles of 6 h–6 h, 8 h–8 h and 12 h–12 h, respectively. The mortality in these cases was much higher than that in the 115% constant TDG test, where the mortality reached only 5%. This is evidence that peak TDG and duration are driving survival.

This study showed that peak TDG is the driver of survival and that fluctuation has little effect. The LT50 of fish in the CT-2 case (peak TDG: 120%) was significantly higher than that in the FT-2 case (peak TDG: 125%) and FT-3 case (peak TDG: 130%). The FT-1(a–c) group and CT-2 group had the same peak TDG (120%), and there was no significant difference in survival between them according to the log-rank Mantel-Cox test. Under the fluctuation range of 115–125%, a significant difference in fish survival was found only between the 6 h–6 h cycle and the 8 h–8 h cycle (*p* = 0.027). The fluctuation cycle did not affect fish survival at the fluctuation range of 110–130%. In Wang’s studies [30,35], fish exposed to 6 h–6 h TDG supersaturation (130–110%), exhibited a longer LT50 than those exposed to 130% TDG supersaturation, but the difference was not significant. Intermittent saturated-equilibrium water did not significantly prolong the survival time of the fish, which is similar to the conclusion of this study.

Due to construction of a large number of world-class high dams on the upper Yangtze River, the problem of TDG supersaturation needs to be solved urgently. At present, the physiological and behavioural responses of fish in the upper Yangtze River to TDG supersaturation are not fully understood, and a system for evaluating the influence of TDG supersaturation has not been systematically established. The research in this paper helps elucidate the responses of rare and endemic fishes in the upper Yangtze River to TDG supersaturation, which provides a reference for the ecological operation of hydropower engineering in China and can also be used for fish protection in other river basins of the world.

## 5. Conclusions

Laboratory and field experiments were carried out with *Schizothorax prenanti* to study the tolerance characteristics of resident fish in the upper Yangtze River under fluctuating of TDG supersaturation, and the following conclusions were drawn:

1. The TDG supersaturation in the natural river changed greatly during the flood period due to reservoir regulation. The TDG peak and the inability to compensate by fleeing to greater depths affect the survival of fish. A high TDG peak and shallow compensatory water depth caused the fish mortality in the field experiments to be higher in September than in July.
2. In the laboratory experiment, the tolerance of the fish under fluctuating TDG supersaturation was lower than that under constant TDG. The fish tolerance of TDG supersaturation varied depending on the TDG peak and duration. Under the fluctuation range of 115–125%, fish survival in the 6 h–6 h cycle was significantly different from that in the 8 h–8 h cycle. The fluctuation cycle did not affect fish survival at the fluctuation range of 110–130%.
3. Intermittent lower TDG supersaturation does not significantly enhance the tolerance of fish.

## Figures and Tables

**Table 1:** Conditions of the fluctuant total dissolved gas (TDG) test. Cases with the same letter have the same cycle, and cases with the same number have the same TDG level.

Case	Low TDG	Cycle for Low TDG	High TDG	Cycle for high TDG
FT-1a	110%	6 h	120%	6 h
FT-1b	110%	8 h	120%	8 h
FT-1c	110%	12 h	120%	12 h
FT-2a	115%	6 h	125%	6 h
FT-2b	115%	8 h	125%	8 h
FT-2c	115%	12 h	125%	12 h
FT-3a	110%	6 h	130%	6 h
FT-3b	110%	8 h	130%	8 h
FT-3c	110%	12 h	130%	12 h

**Table 2:** The mortality and prevalence of typical gas bubble disease (GBD) in fish.

Cases	Mortality	Fish Weight (g)	Fish Length (cm)	Prevalence of GBD		
				Pectoral Fin Bubbles	Dorsal Fin Bubbles	Caudal Fin Bubbles
CT-1	5.0%	11.7	10.3	100%	100%	100%
CT-2	70.0%	13.2 ± 6.0	11.2 ± 2.2	75.6%	71.4%	78.6%
FT-1a	60.0%	11.0 ± 6.3	10.7 ± 2.5	75.0%	83.3%	83.3%
FT-1b	80.0%	11.6 ± 6.7	11 ± 2.5	81.3%	100.0%	87.5%
FT-1c	70.0%	16.4 ± 10.1	12.25 ± 2.9	71.4%	85.7%	71.4%
FT-2a	100.0%	11.8 ± 7.8	11 ± 3.6	75.0%	90.0%	85.0%
FT-2b	100.0%	10.9 ± 5.5	10.6 ± 1.9	85.0%	95.0%	65.0%
FT-2c	100.0%	11.1 ± 5.9	10.5 ± 1.9	60.0%	70.0%	75.0%
FT-3a	100.0%	16.9 ± 6.3	12.05 ± 1.9	60.0%	80.0%	85.0%
FT-3b	100.0%	13.8 ± 7.0	11.3 ± 2.0	85.0%	85.0%	100.0%
FT-3c	100.0%	15.8 ± 9.7	11.7 ± 3.2	70.0%	80.0%	70.0%

**Table 3:** The LT50 of experimental fish in each case.

Case	Fluctuation Range	Cycle Time	Fish Weight (g)	Length (cm)	Percent Survival	LT50 (h)
CT-2			13.2 ± 6.0	11.2 ± 2.2	30%	37.7
FT-1a	110–120%	6 h–6 h	11.0 ± 6.3	10.7 ± 2.5	40%	53.8
FT-1b		8 h–8 h	11.6 ± 6.7	11 ± 2.5	20%	38.2
FT-1c		12 h–12 h	16.4 ± 10.1	12.25 ± 2.9	30%	40.9
FT-2a	115–125%	6 h–6 h	11.8 ± 7.8	11 ± 3.6	0%	13.6
FT-2b		8 h–8 h	10.9 ± 5.5	10.6 ± 1.9	0%	17.6
FT-2c		12 h–12 h	11.1 ± 5.9	10.5 ± 1.9	0%	18.5
FT-3a	110–130%	6 h–6 h	16.9 ± 6.3	12.05 ± 1.9	0%	15.6
FT-3b		8 h–8 h	13.8 ± 7.0	11.3 ± 2.0	0%	19.2
FT-3c		12 h–12 h	15.8 ± 9.7	11.7 ± 3.2	0%	18.3

**Table 4:** Result of the log-rank Mantel–Cox tests between cases.

Case		CT-2	FT-1a	FT-1b	FT-1c	FT-2a	FT-2b	FT-2c	FT-3a	FT-3b	FT-3c
**CT-2**	*χ* ^2^										
	*P*										
FT-1a	*χ* ^2^	0.854									
	*P*	0.355									
FT-1b	*χ* ^2^	0.062	1.342								
	*P*	0.804	0.247								
FT-1c	*χ* ^2^	0.008	0.538	0.189							
	*P*	0.927	0.463	0.664							
FT-2a	*χ* ^2^	18.344	33.325	19.146	28.537						
	*P*	0.000	0.000	0.000	0.000						
FT-2b	*χ* ^2^	11.407	21.059	13.515	13.923	4.912					
	*P*	0.001	0.000	0.000	0.000	0.027					
FT-2c	*χ* ^2^	13.456	26.609	14.333	26.490	1.910	0.454				
	*P*	0.000	0.000	0.000	0.000	0.167	0.501				
FT-3a	*χ* ^2^	15.920	29.363	15.778	27.560	0.001	1.925	1.307			
	*P*	0.000	0.000	0.000	0.000	0.978	0.165	0.253			
FT-3b	*χ* ^2^	12.120	24.637	12.852	24.833	12.774	0.355	4.615	7.361		
	*P*	0.000	0.000	0.000	0.000	0.000	0.551	0.032	0.007		
FT-3c	*χ* ^2^	12.576	25.591	14.672	25.591	0.059	0.179	0.035	0.020	3.469	
	*P*	0.000	0.000	0.000	0.000	0.808	0.672	0.851	0.888	0.063	

**Table 5:** Previous studies on the tolerance of fish in the Yangtze River to TDG supersaturation. Some data are not given in the table because they were not provided in previous studies.

Species	Length (cm)	Weight (g)	Age	T (°C)	LT50 (h)		
					120%	125%	130%
Silver carp [39]	6.2 ± 0.4	5.3 ± 0.6		22.0 ± 0.5			12.04
Rock carp [12,35,39]	7.9 ± 0.5	7.8 ± 0.6		22.0 ± 0.5			6.78
	6.6–7.2	2.4–2.5		25	18.7	15.4	8.2
	9.2 ± 0.9	8.1 ± 0.8	Yearling	21–23	14.6	7.8	4.7
	5.5 ± 0.6	3.3 ± 0.9	Yearling	22 ± 0.6	11.14	6.39	4.32
Chinese sucker [37]	11.5 ± 0.9	39.5 ± 7.6	6 months old	22 ± 0.6	49.06	9.19	7.81
David’s Schizothoracin [36]			7 days old	20 ± 0.5			35.38
Prenant’s Schizothoracin [30,33,34,35]	5.9–9.1	3.1–8.2	Yearling	20 ± 0.5	21.45	16.75	15.3
	6.2 ± 1.0	4.5 ± 1.7	Yearling	22 ± 0.6		19.14	13.67
	14 ± 2	50 ± 18	2 years old	20	10.7	9.5	6.5
	6.0 ± 0.6	4.0 ± 1.3	Yearling	20	23.88	9.66	8.74
